# Hypercholesterolemia Is the Only Risk Factor Consistently Associated with Coronary Calcification in Three European Countries—Euro CCAD Study †

**DOI:** 10.3390/diagnostics15212789

**Published:** 2025-11-04

**Authors:** Artan Bajraktari, Ibadete Bytyçi, Axel Diederichsen, Axel Schmermund, Michael Y. Henein

**Affiliations:** 1 Institute of Public Health and Clinical Medicine, Umea University, 901 87 Umea, Sweden; ibadete.bytyci@uni-pr.edu; 2 Clinic of Cardiology, University Clinical Centre of Kosovo, 10000 Prishtina, Kosovo; 3 Syddansk Universitet, 5230 Odense, Denmark; axel.diederichsen@rsyd.dk; 4 Bethanien Hospital, 63089 Frankfurt, Germany; a.schmermund@ccb.de; 5 Imperial College London, London SW7 2AZ, UK; henein@gmail.com

**Keywords:** coronary artery calcium score, hypercholesterolemia, atherosclerosis, coronary artery disease

## Abstract

**Background and Aim:** Coronary calcification has been described as a manifestation of subclinical atherosclerosis. However, its predictors are not well established. The aim of this study was to evaluate the relationship between coronary artery calcium score (CACs) evaluated by multi-detector computed tomography (MDCT) and atherosclerotic cardiovascular disease (CVD) risk factors in symptomatic patients in three European countries. **Method:** We retrospectively analyzed 550 patients (age 62.7 ± 12 year, 47.5% females) who presented with atypical chest pain in Germany, Denmark, and Sweden. The demographic indices, CVD risk factors, and CACs were analyzed. The CV risk factors were classified as low (no risk factors), intermediate (1–2 risk factors), and high (≥3 risk factors). Patients were geographically classified into: Gr. I–German (n = 344), Gr. II–Danish (n = 84), and Gr. III–Swedish (n = 122) patients. **Results:** In the cohort as a whole, the mean CACs was 270.3 ± 72, and the intermediate risk was more prevalent than low and high-risk (*p* < 0.05 for all). Among the CVD risk profile, arterial hypertension (AH) was the most prevalent, followed by hypercholesterolemia, obesity, smoking, and diabetes (53, 38.2, 23.7, 17.6, and 10.5%; *p* < 0.05 for all). The German population was younger and had less CVD risk factors compared to the Danish and Swedish populations (*p* < 0.05, for all). CACSs adjusted to age and sex was lowest in Swedish patients, followed by German patients, and highest in Danish patients (*p* < 0.05). The CACs modestly correlated with age (r_pb_ = 0.52, *p* < 0.001), sex (r_pb_ = 0.48, *p* < 0.001), and extent of risk (r_pb_ = 0.35, *p* = 0.001). On multivariate regression analysis, hypercholesterolemia β = 185.1 (63.11 to 307.1), the extent of risk adjusted for age and sex β 3.741 (2.566 to 4.916; *p* < 0.001), and AH, β = 142.6 (11.25 to 274.1; *p* = 0.03) independently correlated with CACs. Furthermore, hypercholesterolemia was the only risk factor, consistently associated with CACs across all three countries. **Conclusions:** In symptomatic European patients, hypercholesterolemia is the main player in coronary calcium formation.

## 1. Introduction

The prevention of acute coronary and cerebrovascular (CV) syndromes relies on accurate identification of patients at risk for developing such events [1]. Despite the beneficial use of different available scores and models for identifying such individuals who carry risk for acute events, many of them fall outside the published prediction tables [2,3]. Coronary calcification has been described as a manifestation of subclinical atherosclerosis [4] and its accurate measurement [calcium score (CACs)] made it a part of the routine tools for assessing patients at intermediate risk for coronary artery disease, particularly those presenting with atypical symptoms. CACs is now widely regarded as a valuable tool, offering substantial additional insight into the severity and extent of coronary atherosclerosis, over and above conventional risk factors, particularly in its relationship with symptoms [5]. Numerous studies have delved into the relationship between coronary events, atherosclerosis metrics, and CACs [6,7,8,9]. Villines et al. found that a CACs of zero yielded a 99% predictive value for detecting coronary stenosis greater than 70% [10]. Lamonte et al. demonstrated a direct link between CACs and the incidence of coronary heart disease (CHD) events in asymptomatic individuals, highlighting CACs as a significant predictor for all CHD events [6]. Furthermore, the Rotterdam Study, in 2000 patients, showed that CACs led to significant risk reclassification, particularly among individuals initially categorized at intermediate risk according to the Framingham Risk Score [7]. In addition, a milestone in the validation of CACs came from the MESA study, which decisively established its superiority over carotid intimal medial thickness [11]. Despite such impact of CACs in classifying individuals, its interpretation can be influenced by patient characteristics including age, gender, and ethnicity [12]. We hypothesized that the prevalence and severity of CAC would vary between European populations due to differences in the distribution of conventional cardiovascular risk factors, and that hypercholesterolemia likely has a consistent association with CAC across different countries. The aim of this study was to evaluate the relationship between CACs and CVD risk factors in symptomatic patients in three European countries, Germany, Denmark, and Sweden.

A preliminary version of this study was presented as a conference abstract at Atherosclerosis 2024 [13].

## 2. Methods

### 2.1. Study Design and Patients

**Figure 1:**
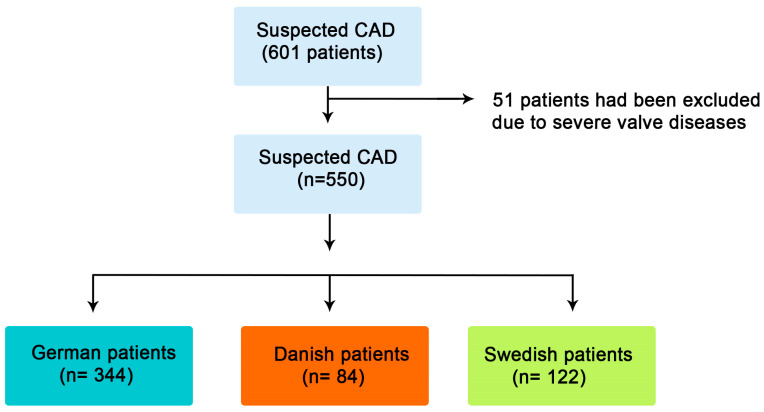
Flow chart of included patients.

This study is a cross-sectional retrospective analysis of a cohort of 550 patients with atypical chest pain, chest discomfort that is not typical for coronary artery disease, collected from four heart centers in three countries (Germany, Denmark, and Sweden). All patients were suspected of having CAD. Patients with previous history or evidence for acute coronary syndrome (ACS), heart failure, or more than mild valve disease were excluded from the study. Based on the country of origin, patients were classified into: Gr. I–German patients (n = 344), Gr. II–Danish patients (n = 84), and Gr. III-Swedish patients (n = 122) (Figure 1). The study was conducted in accordance with the national and institutional guidelines as well as the revised Helinski Declaration (Dnr: 08-118M; J. nr. H-B-2009-027; approved on 15 March 2009).

### 2.2. Cardiovascular Risk Factor Assessment

Cardiovascular risk factors, including arterial hypertension (AH), type 2 diabetes mellitus (DM), hypercholesterolemia, obesity, and smoking were evaluated using medical records and prior assessments and treatments. In accordance with the established international criteria and body mass index (BMI) cutoff values, individuals with a BMI of 25–29.9 kg/m^2^ were categorized as overweight, and those with a BMI of ≥30 kg/m^2^ as individuals living with obesity. Systemic AH was diagnosed when systolic blood pressure (SBP) was ≥140 mmHg and/or diastolic blood pressure (DBP) was ≥ 90 mmHg, or the patients were under the treatment for AH. Type 2 diabetes mellitus (DM) was identified based on pre-recruitment diagnoses that led to the initiation of conventional oral hypoglycemic and/or insulin therapy. Hypercholesterolemia was defined as a total cholesterol level of >5.0 mmol/L (193 mg/dl) or the use of lipid lowering medications. The cardiovascular atherosclerosis risk factors were stratified into three categories: low (no risk factor), intermediate (1–2 risk factors), and high (≥3 risk factors) [5].

### 2.3. Coronary Artery Calcium (CACs)

Coronary artery calcium (CAC) assessment was conducted using a 64-slice multidetector computed tomography (MDCT) scanner (Somatom Sensation Cardiac 64; Siemens Medical Solutions, Forchheim, Germany) with a gantry rotation time of 330 milliseconds (collimation 64 × 0.6 mm, reconstruction increment 0.3 mm). The Swedish cohort was studied by 64-MDCT (LightSpeed VCT, GE Healthcare, Milwaukee, WI, USA) and the Danish cohort using 64-MDCT (GE 64-slice scanner or Siemens Flash). In all participants, image acquisition took place during a quiet expiratory pause. When resting heart rate exceeded 60 beats per minute, oral beta-blockers (specifically, bisoprolol 5 mg or metoprolol 50 mg) were administered an hour prior to the scan. Calcification was defined as the presence of more than two contiguous pixels with a density exceeding 130 Hounsfield Units. Specialized workstation software automatically identified and highlighted these calcified areas in color. The individual lesion scores were then automatically summed to calculate the total Agatston score for the entire coronary tree [14]. Based on CACs, patients were classified into very low (0–10), low (11–100), intermediate (101–400), and severe (>400) coronary calcification.

### 2.4. Statistical Analysis

Data are summarized using frequencies (percentages) for categorical variables and mean ± standard deviation (SD) for continuous variables or median interquartile (IQR) ranges. Continuous data were compared using a two-tailed Student’s *t*-test and discrete data were assessed using the Chi-square test. When comparing quantitative variables among more than two groups, analysis of variance (ANOVA) and Bonferroni statistical tests were applied. To assess the degree of association between CACs and demographic indices, we used Pearson’s correlation coefficient in the case of continuous variables. The Chi-square test (categorical variables) and point-biserial correlation was used for categorical and continuous variables. Because the impact of age and sex on CACs is well established, we used the general linear model to compare age- and sex-adjusted CACs between groups.

The regression analyses were conducted to identify independent associations between risk factors and CACs. Associates of increased CACs were identified using both univariate and multivariate analyses. We initially ran univariate analyses for each risk factor (age, sex, hypertension, diabetes mellitus, hypercholesterolemia, obesity, smoking, and total number of risk factors) followed by a multivariate model adjusted for age and gender to identify independent associations. A significant difference was defined as a *p*-value of <0.05 (two-tailed). All statistical analyses were conducted using SPSS Software Package version 26.0 (IBM Corp., Armonk, NY, USA).

## 3. Results

### 3.1. Demographic Indices

The mean age of patients was 61.9 ± 12 years, and the mean CACs was 270.3 ± 72 AU (mean ± SD). A relatively small proportion, only 17.3%, were less than 50 years of age, and 49.3% of the patients were female (Table 1).

### 3.2. Impact of CV Risk Factors on CACs

**Figure 2:**
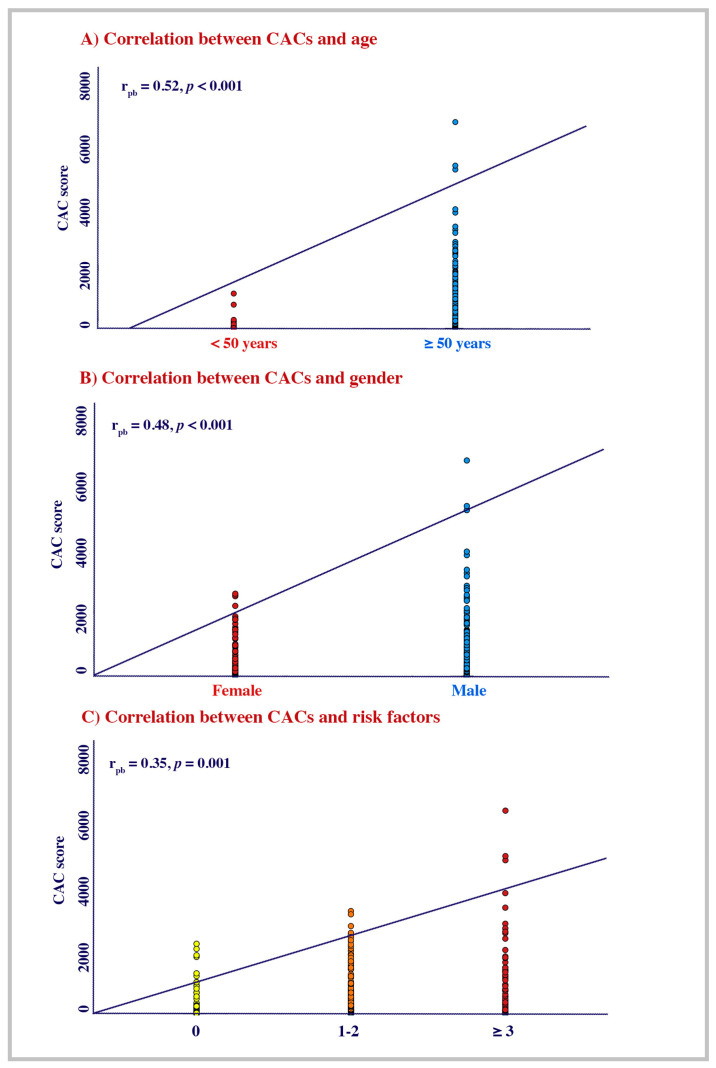
Correlation of mean CACs with age, gender, and CV risk factors.

Three hundred and twenty-two (53.5%) patients were on antihypertensive treatment and fifty-eight (10.8%) were diagnosed with DM. A total of 203 (38.2%) patients had hypercholesterolemia, 130 (23.7%) were participants with obesity, and 97 (17.6%) were current smokers. Out of the whole cohort, 184 (36.6%) were on statins. Patients with intermediate CV risk were more prevalent compared to those with low and high CV risk (62.9%, 19.5%, 17.3%; *p* < 0.001; Table 1). The very low CACs were the most prevalent among the cohort while the low CACs, intermediate CACs, and the severe CACs were almost similar (45.6%, 16.5%, 15.8, and 22.2%; *p* < 0.001). The CACs modestly correlated with age (r_pb_ = 0.51, *p* < 0.001), sex (r_pb_ = 0.48, *p* < 0.001), and the level of CV atherosclerosis risk (r_pb_ = 0.35, *p* = 0.001; Figure 2A–C).

### 3.3. The Relationship Between Ethnicity, CV Risk, and CACs

**Figure 3:**
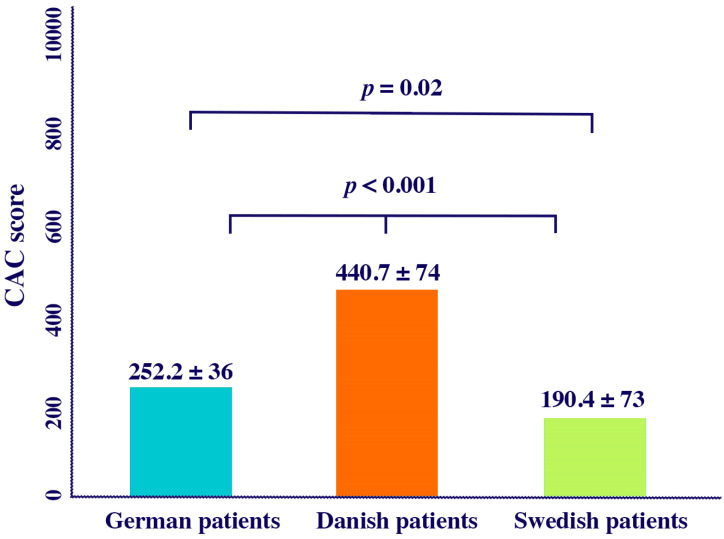
Mean CACs adjusted by age in different countries.

German patients were younger compared to Danish and Swedish patients. Females were more prevalent among the Danish patients compared to the Swedish and the German patients (*p* < 0.05, for all). In contrast, the Swedish patients had more intermediate risk factors and fewer smokers compared to the other two ethnic groups (*p* < 0.05, for all; Table 2). The mean CACs adjusted for age and sex, was lower in Swedish patients compared to the German and the Danish patients without AS (190.4 ± 73, 252.2 ± 36, 440.7 ± 74; *p* < 0.05 for all) (Figure 3).

### 3.4. Correlates with CACs

In univariate analysis, AH (*p* = 0.01), DM (*p* = 0.04), hypercholesterolemia (*p* < 0.001), smoking (*p* = 0.01), and CV risk factors (*p* < 0.001) correlated with CACs but in the multivariate analysis, the independent associates of CACs proved to be CV risk factors adjusted to age and sex β = 3.741 (2.566 to 4.916; *p* < 0.001), hypercholesterolemia β = 185.1 (63.11 to 307.1; *p* = 0.003), and AH β = 142.6 (11.25 to 274.1; *p* = 0.03) (Table 3a). In a sub-analysis trying to identify associates of CACs in different countries, hypercholesterolemia was the independent associate in the three groups of patients; German [(OR = 149.5 (47.13 to 252.1); *p* = 0.01)], Swedish [(198.6 (0.904.7 to 601.9) *p* = 0.043)], and Danish OR = 542.1 (14.29 to 961.1; *p* = 0.031) patients. Likewise, the extent of atherosclerosis risk factors adjusted for age and sex independently associated CACs in German and Swedish patients but not in Danish patients. AH tended to be associated with CACs in German, Danish, and Swedish patients (*p* = 0.073, *p* = 0.056 and *p* = 0.061, respectively) but did not reach statistical significance (Table 3b).

## 4. Discussion

The results of this cohort of symptomatic patients at intermediate risk for significant coronary artery disease revealed the following: (1) the German population were younger and with lower cardiovascular risk when compared to the Danish and Swedish populations; (2) a linear relationship was observed between the presence of CACs and the number of atherosclerosis risk factors; (3) after adjusting for age and sex, the Swedish patients proved to have the lowest CACs, followed by German patients; and (4) hypercholesterolemia adjusted for age and sex, was the main independent associate with CACs, irrespective of country, on multivariate analysis.

Many studies have investigated the role of CACs in coronary events in symptomatic and asymptomatic patients [6,7,12,15]. Studies have also shown relationships between conventional atherosclerosis risk factors and CACs with varying strength, and with DM being the main player [16,17] as well as obesity. Although atherosclerosis risk factors are known to contribute to the development of coronary artery disease with the highest impact being their accumulative risk score, CACs has been proposed as a risk factor for coronary stenosis, on its own merit [4]. The direct relationship between CACs and severity of coronary stenosis is now well established, although almost 10% of cohorts fall outside the bell curve with some having zero CAC but severe stenosis and others extensive calcification but no flow limiting lesions [18,19]. Our results adhere with the above findings in ascertaining a relationship between CACs and risk factors, in particular hypercholesterolemia and hypertension, although only in a modest way, particularly in aged men compared to young females. These findings are supported by previous publications which showed a very small percentage of women (3%) exhibited a CACs above zero at the age of 40 years [20,21,22]. Furthermore, it seems that ethnicity plays an important role which could add to the interpretation of our findings. After adjusting for demographic indices, mean CACs proved lower in the Swedish population compared to the German and Danish populations. Despite that, hypercholesterolemia proved to be the main independent associate of CACs in the three countries. Such consistent finding must have significant clinical implications, through better CAD prevention. CAC is well recognized as a form of subclinical atherosclerosis in patients at intermediate risk of coronary disease. It has also been shown to provide objective evidence of the healing of coronary inflammation, with CACs increasing in patients treated with high-dose statins [23,24,25]. Thus, while CAC is an eye opener for the optimal management of CV atherosclerosis in accordance with guidelines, its increase in severity over time, with statins, should be seen as a sign of disease stability and reduced vulnerability. In our cohort the lowest prevalence of patients with hypercholesterolemia and those taking statins was observed among Germans, compared to the Danes and the Swedes.

Our findings highlight some demographic and risk factor differences between the populations of symptomatic patients in Europe. However, after correction for age and sex, hypercholesterolemia was singled out as the main associate with CACs, irrespective of the country, thus highlighting its serious impact on atherosclerosis. Arterial hypertension and the accumulative number of risk factors differed between countries, thus respective patients need individualistic cardiovascular disease prevention strategies, depending on each individual’s risk factors. Although our study focused exclusively on CACs as a marker of subclinical atherosclerosis, recent evidence highlights the pericoronary fat attenuation index (pFAI) as a complementary imaging biomarker that reflects local coronary inflammation. Incorporating both CACs and pFAI in future studies could provide a more comprehensive assessment of coronary risk and plaque vulnerability [26].

Limitations: This study has some limitations. First, we did not attempt a direct comparison of the role of CACs in conjunction with conventional risk factors to predict significant coronary artery disease, since the objective was to identify the relevance of individual and combined risk factors in predicting CACs. Second, this study is based on cross-sectional data, which provides only a single-time snapshot of patient characteristics and CACs. Therefore, our findings reflect associations rather than causal relationships, as we cannot determine the temporal sequence or duration of exposure to individual risk factors that may have influenced CAC development. Future longitudinal or panel studies, including repeated CAC assessments over time, are needed to clarify how specific risk factors, such as diabetes mellitus, hypertension, and hypercholesterolemia, contribute to the progression of coronary calcification. Third, the unequal distribution of participants across the three national cohorts and smaller numbers in some subgroups may have impacted the statistical power of detecting associations. Future studies with larger and more balanced sample sizes across populations would allow for more robust between-group comparisons. Finally, genetic loci markers, including lipoprotein (a) and epicardial coronary fat were not evaluated in this study; they could have had an impact on the results and conclusion.

Conclusions: The findings of this study underscore the significant role of hypercholesterolemia in the formation of coronary calcium irrespective of the European country, hence the need for optimum treatment of hypercholesterolemia to combat its known complications.

## Figures and Tables

**Table 1:** Demographic and clinical data of patients.

Variable	Patients
	(n = 550)
Demographic and clinical data	
Age (years)	61.9 ± 12
Age < 50 years (n, %)	95 (17.3)
Females (n, %)	271 (49.3)
BMI (m/kg^2^)	27.1 ± 4.1
SBP (mmHg)	144 ± 18
DBP (mmHg)	83 ± 10
Individuals living with obesity (n, %)	130 (23.7)
AH (n, %)	322 (53.5)
DM (n, %)	58 (10.5)
Hypercholesterolemia (n, %)	203 (38.2)
Statins (n, %)	184 (36.6)
Smoking (n, %)	97 (17.6)
Number of risk factors	
0 risk factor (n, %)	107 (19.5)
1–2 risk factor (n, %)	346 (62.9)
≥3 risk factors (n, %)	95 (17.3)
Calcium scoring	270.3 ± 72
Very low (0–10), (n, %)	252 (45.6)
Low (11–100), (n, %)	91 (16.5)
Intermediate (101–400), (n, %)	87 (15.8)
Severe (>400), (n, %)	122 (22.2)

AH: arterial hypertension; BMI: body mass index; DM: diabetes mellitus; SBP: systolic blood pressure; DBP: diastolic blood pressure. Data are presented as mean ± SD or percentage.

**Table 2:** Demographic and clinical data of patients in different regions.

Variable	German	Danish	Swedish	*p*
	Patients	Patients	Patients	Value
	(n = 84)	(n = 344)	(n = 122)	
Demographic and clinical data				
Age (years)	56.7 ± 10 ^a*,b*^	71.6 ± 6.7	69.8 ± 9.3	<0.001
Age < 50 years (n, %)	93 (27) ^a*,b*^	0 (0) ^c*^	1 (0.81)	<0.001
Females (n, %)	175 (50.8) ^a*,b*^	29 (34.5)	67 (54.9)	<0.001
BMI (m/kg^2^)	26.9 ± 4.2	27.1 ± 4.1	27.3 ± 4.1	NS
SBP (mmHg)	140 ± 17	145 ± 19	143 ± 18	NS
DBP (mmHg)	84 ± 11	86 ± 11	78 ± 9.3	NS
Obesity (n, %)	80 (24.4)	19 (22.4)	31 (25.4)	NS
AH (n, %)	138 (40.1) ^a*^	62 (73.8) ^c*^	88 (72.1)	0.04
DM (n, %)	21 (6.10)	16 (19.1)	22 (18.0)	0.03
Hypercholesterolemia (n, %)	68 (19.8) ^a*^	54 (64.2) ^c*^	82 (67.2)	0.001
Statins (n, %)	92 (26.7) ^a*,b^	44 (51.2) ^c*^	78 (64)	<0.001
Smoking (n, %)	79 (22.9) ^a*,b^	11 (13.1) ^c*^	8 (6.55)	<0.001
Number of risk factors				
0 risk factor (n, %)	90 (26.1) ^a*,b^	17 (20.2) ^c*^	9 (7.37)	0.004
1–2 risk factor (n, %)	218 (63.4) ^a*,b^	42 (50.0) ^c*^	90 (73.3)	<0.001
≥3 risk factors (n, %)	36 (10.5) ^a*,b^	25 (29.7) ^c^	23 (18.8)	0.02
Calcium score	252.2 ± 36 ^a*^	440.7 ± 74 ^c^	190.4 ± 73	0.001
Very low (0–10 AU)	214 (62.2) ^a*,b^	13 (15.5) ^c*^	37 (30.3)	
Low (11–100 AU)	60 (17.4) ^a^	10 (11.9) ^c^	20 (16.4)	0.02
Intermediate (101–400 AU)	37 (10.8) ^a,b^	21 (25.0) ^c^	29 (32.8)	NS
Severe (>400 AU)	33 (9.59) ^a*,b*^	41 (48.8) ^c*^	36 (29.5)	NS

(a) *p* < 0.05; Gr. I vs. II; (b) *p* < 0.05; Gr. I vs. III; (c) *p* < 0.05; II vs. III; (a*) *p* < 0.001; Gr. I vs. II; (b*) *p* < 0.001; Gr. I vs. III; (c*) *p* < 0.001; II vs. III; AH: arterial hypertension; BMI: body mass index; DM: diabetes mellitus; SBP: systolic blood pressure; DBP: diastolic blood pressure; AU: Agatston units. Data are presented as mean ± SD or percentage.

**Table 3:** (**a**) Univariate associates of CACs, (**b**) Multivariate associates of CACs.

(**a**)		
**Variable**	**Univariate Associates**	***P***
	**β (95% CI)**	**Value**
	All patients	
Diabetes mellitus	64.89 (11.02 to 195.3)	0.041
Arterial hypertension	236.1 (101.1 to 371.1)	0.011
Hypercholesterolemia	168.1 (109.1 to 360.2)	<0.001
Obesity	18.87 (−129.5 to 167.2)	0.053
Smoking	−110.1 (−268.3 to 48.22)	0.013
Risk factors *	36.07 (11.12 to 121.4)	<0.001
	German patients	
Diabetes mellitus	222.3 (106,8 to 700.1)	0.291
Arterial hypertension	207.1 (1.699 to 544.6)	0.026
Hypercholesterolemia	118.1 (33.99 to 241.1)	0.001
Obesity	6.351 (−19.11 to 5.172)	0.211
Smoking	−33.11 (−88.44 to 109.2)	0.301
Risk factors *	2.204 (0.330 to 2.009)	<0.001
	Danish patients	
Diabetes mellitus	321.1 (−1.291 to 721.3)	0.331
Arterial hypertension	221.3 (0.951 to 538.9)	0.022
Hypercholesterolemia	331.1 (104.2 to 759.3)	0.001
Obesity	99.21 (−0.125 to 251.1)	0.115
Smoking	−102.3 (−312.4 to 291.1)	0.211
Risk factors *	3.131 (0.121 to 2.89)	0.01
	Swedish patients	
Diabetes mellitus	151.1 (−33.12 to 405.0)	0.661
Arterial hypertension	177.1 (0.978 to 272.8)	0.021
Hypercholesterolemia	188.2 (104.1 to 522.1)	0.022
Obesity	7.110 (−1.118 to 99.21)	0.331
Smoking	222.5 (−188.1 to 516.0)	0.492
Risk factors *	3.101 (1.088 to 6.219)	<0.001
(**b**)		
Variable	Multivariate Associates	*P*
	β (95% CI)	Value
	All patients	
Diabetes mellitus	−124.4 (−314.8 to 65.89)	0.201
Arterial hypertension	142.6 (11.25 to 274.1)	0.030
Hypercholesterolemia	185.1 (63.11 to 307.1)	0.003
Obesity	−32.88 (−170.9 to 105.1)	0.442
Smoking	−163.9 (−311.0 to −16.82)	0.073
Risk factors *	3.741 (2.566 to 4.916)	<0.001
	German patients	
Diabetes mellitus	156.8 (−15.68 to 630.6)	0.311
Arterial hypertension	144 (−0.168 to 258.8)	0.073
Hypercholesterolemia	149.5 (47.13 to 252.1)	0.011
Obesity	2.055 (−82.08 to 7.972)	0.410
Smoking	−59.17 (−156.4 to 38.11)	0.230
Risk factors *	1.179 (0.465 to 1.893)	0.001
	Danish patients	
Diabetes mellitus	542.1 (14.29 to 961.1)	0.281
Arterial hypertension	393.9 (−12.51 to 800.4)	0.056
Hypercholesterolemia	542.1 (14.29 to 961.1)	0.031
Obesity	27.09 (−12.24 to 66.43)	0.172
Smoking	−377.3 (−856.4 to 101.6)	0.122
Risk factors *	1.22 (−0.8823 to 3.336)	0.254
	Swedish patients	
Diabetes mellitus	58.92 (267.2 to 385.0)	0.720
Arterial hypertension	224.4 (−37.01 to 485.8)	0.061
Hypercholesterolemia	198.6 (094.7 to 601.9)	0.043
Obesity	4.430 (24.18 to 33.04)	0.751
Smoking	213.4 (−613.8 to 186.8)	0.292
Risk factors *	2.203 (0.717 to 3.689)	0.004

* Adjusted by age and gender.

## Data Availability

The data underlying this article will be shared at reasonable request to the corresponding author.
